# Early Antiinflammatory Therapy Attenuates Brain Damage After Sah in Rats

**DOI:** 10.1515/tnsci-2019-0018

**Published:** 2019-04-23

**Authors:** Georg Vadokas, Stefan Koehler, Judith Weiland, Nadine Lilla, Christian Stetter, Thomas Westermaier

**Affiliations:** 1 Department of Neurosurgery, University Hospital Würzburg, Josef-Schneider-Straße 11, 97080 Würzburg, Germany; 2 Department of Urology, Canisius Wilhelmina Hospital Nijmegen, Weg door Jonkerbos 100, 6532 SZ Nijmegen, Netherlands

**Keywords:** subarachnoid hemorrhage, early brain injury, methylprednisolone, minocycline, neuroprotection

## Abstract

**Background:**

Early inflammatory processes may play an important role in the development of early brain injury (EBI) after subarachnoid hemorrhage (SAH). Experimental studies suggest that anti-inflammatory and membrane-stabilizing drugs might have beneficial effects, although the underlying mechanisms are not fully understood. The aim of this study was to investigate the effect of early treatment with methylprednisolone and minocycline on cerebral perfusion and EBI after experimental SAH.

**Methods:**

Male Sprague-Dawley rats were subjected to SAH using the endovascular filament model. 30 minutes after SAH, they were randomly assigned to receive an intravenous injection of methylprednisolone (16mg/kg body weight, n=10), minocycline (45mg/kg body weight, n=10) or saline (n=11). Mean arterial blood pressure (MABP), intracranial pressure (ICP) and local cerebral blood flow (LCBF) over both hemispheres were recorded continuously for three hours following SAH. Neurological assessment was performed after 24 hours. Hippocampal damage was analyzed by immunohistochemical staining (caspase 3).

**Results:**

Treatment with methylprednisolone or minocycline did not result in a significant improvement of MABP, ICP or LCBF. Animals of both treatment groups showed a non-significant trend to better neurological recovery compared to animals of the control group. Mortality was reduced and hippocampal damage significantly attenuated in both methylprednisolone and minocycline treated animals.

**Conclusion:**

The results of this study suggest that inflammatory processes may play an important role in the pathophysiology of EBI after SAH. Early treatment with the anti-inflammatory drugs methylprednisolone or minocycline in the acute phase of SAH has the potential to reduce brain damage and exert a neuroprotective effect.

## Introduction

The pathophysiology and treatment of early brain injury (EBI) have been more intensively focussed since drugs that successfully treated delayed cerebral vasospasm (DCV) failed to improve the neurological outcome of patients who suffered aneurysmal subarachnoid hemorrhage (SAH) 1,2. The initial presentation, as documented by the Hunt/Hess and WFNS classifications, is still one of the most powerful predictors of long-term outcome. This further points out the importance of EBI 3,4. Furthermore, it cannot be excluded that early pathophysiological changes may significantly influence mechanisms of delayed neurological deterioration occurring later in the course of the disease 5,6.

Apart from impaired cerebral perfusion, inflammatory pathways have been reported to contribute to EBI 7,8. It is assumed that the accumulation of hemoglobin in the subarachnoid space and contact with collagen fibers trigger an immunological reaction [7], resulting in the activation of immunomodulators and, finally, diapedesis of leukocytes and macrophages into the brain parenchyma. Degranulation of macrophages, in turn, may result in an amplification of immunological cascades as well as the formation of reactive oxygen species, damaging neurons and glial cells and cerebral blood vessels and thus causing necrotic and apoptotic cell death 9.

Because of its effect on the glucocorticoid receptor, methylprednisolone (MTP) interferes with the production and effect of inflammatory cytokines, chemokines and cell-adhesion molecules 10. In addition, glucocorticoids could also target pathomechanisms which inhibit the prostaglandin synthesis early after SAH and cause early vasospasm 11. Recently Gomis et al. reported the results of a randomized controlled clinical trial, that patients who were treated with MTP after SAH showed significantly better clinical recovery one year after SAH 12.

Minocycline (MC) is a tetracycline antibiotic with the potential to inhibit inflammatory pathways occurring early after SAH. A neuroprotective potential has been observed in neurodegenerative diseases and traumatic brain injury 13,14. MC is a lipophilic drug and able to pass the blood brain barrier 15. It exerts a number of anti-inflammatory mechanisms and may ameliorate the toxic effects of free subarachnoid hemoglobin, which is likely to be a trigger of inflammation after SAH 14,16. It was the aim of this study to investigate the neuroprotective effects of MTP and MC on EBI after experimental SAH.

## Material and Methods

For the experiments, 35 male Sprague-Dawley rats (purchased from Charles River, Sulzfeld, Germany) with a body weight between 270g and 370g were used. All experiments were approved by the regional authorities and the district government of Bavaria, Germany.

### Anesthesia and monitoring

The rats were anesthetized with 4% Isoflurane (Isoflurane CP, CP-Pharma Handelsges. mbH, Burgdorf, Germany), orally intubated and mechanically ventilated with an air/oxygen/Isoflurane mixture. After induction of anesthesia, Isoflurane was reduced to 2% for surgical procedures and the following monitoring interval. Brain temperature was measured throughout the experiment using a temporalis muscle probe. With a heating lamp the temperature was kept at 37°C throughout the experiment. A polyethylene catheter (Portex® 0,58x 0,96mm, Smiths Medical, Kent, UK) was placed into the tail artery for continuous measurement of mean arterial blood pressure (MABP) and blood sampling. Arterial blood gases were measured 30 minutes and 5 minutes before induction of SAH and in hourly intervals thereafter.

### Intracranial pressure and local cerebral blood flow

To measure the intracranial pressure (ICP), a burr hole was drilled over the right frontal cortex 3 mm lateral and 0.5 mm anterior of the bregma. The dura mater was opened diathermically. Two further burr holes were drilled 2 mm posterior and 5 mm lateral on each side of the bregma for the measurement of the local cerebral blood flow (LCBF) via laser-Doppler flowmetry (LDF). Care was taken not to injure the dura mater. After all burr holes were completed, the animals were placed in a supine position with their head fixed in a stereotactic frame with non-rupture ear bars. A Camino ICP-probe (Integra Neurosciences, Plainsboro, NJ, USA) was advanced 2 mm into the brain using a micromanipulator. Using two further micromanipulators, rectangularly bent laser-Doppler probes were positioned in the posterior burr holes, continuously measuring LCBF bilaterally in the area of the cerebral cortex supplied by the middle cerebral artery. The signal was recorded by a two-channel laser-Doppler flowmeter (MBF3D, Moor Instruments, Axminster, England).

### Endovascular vessel perforation – induction of SAH

SAH was caused by applying the endovascular puncture technique, introduced by Bederson et al. 17. After surgical exposure of the right cervical carotid bifurcation, temporary aneurysm clips were positioned on the common and internal carotid artery. A 3-0 Prolene© filament (Ethicon, Inc., Somerville, NJ, USA) was inserted into the external carotid artery and fixed with a silk ligature. The aneurysm clips were removed and the filament was advanced into the internal carotid artery until ipsilateral LCBF decreased, indicating occlusion of the middle cerebral artery by the filament. The suture was then advanced an additional 2-3 mm for intracranial vessel perforation and then quickly withdrawn into the external carotid artery to allow instantaneous reperfusion of the internal carotid artery and development of SAH. SAH was confirmed by a rapid bilateral decrease of LCBF and increase of ICP.

### Experimental groups and intervention

Four animals died or were sacrificed before the randomization process due to irregularities in the induction of anesthesia (n = 2) or endotracheal intubation (n = 2). The remaining 31 animals were randomly assigned to one of the following groups:

1. Methylprednisolone (MTP) group (n=10): intraperitoneal injection with 16 mg/kg body weight MTP (Urbason® solubile, Sanofi-Aventis Germany GmbH, Frankfurt am Main), 30 minutes after SAH.
2. Minocycline (MC) group (n=10): intraperitoneal injection of 45 mg/kg body weight MC (Minocycline Hydrochloride, Sigma Aldrich, Darmstadt, Germany), 30 minutes after SAH.
3. Control group (n=11): intraperitoneal injection of 1 ml/kg body weight saline (B.Braun, Melsungen, Germany), 30 minutes after SAH

At the end of the three-hour monitoring period all probes were removed and the wounds were closed with skin sutures. Anesthesia was withdrawn, analgesia was applied in form of 40 mg/kg body weight metamizole (Novaminsulfon®-ratiopharm, Ratiopharm, Ulm, Germany) intraperitoneally and the rats placed into their home cages.

### Neurological assessment

24 hours after SAH the animals underwent neurological testing including the assessment of hemiparesis and activity. The examinations were carried out by an examiner blinded to the animal’s treatment arm. For neurological examination the animal was placed into an uncovered cage. After a ten-minute acclimatization interval, activity was evaluated by following a previously described observational protocol 18. This protocol resembles a modification of the assessment of spontaneous activity reported by Garcia et al. in the framework of a large-scale assessment protocol for evaluation of the neurological performance after experimental stroke.^19^. In brief, the animal was placed into a large uncovered cage enriched with several stimuli (paper towels, wood tunnel, cardboard box and food pellets) together with a non-operated animal. After 10 minutes of acclimatization, its activity was evaluated after repeated manipulation (tail-holding, lateral push, repeated displacement of the animal). Activity was graded following a 5-grade scale: 4) normal spontaneous activity, 3) slightly reduced spontaneous activity, 2) little or no spontaneous activity, but reaction on stimulus, 1) no activity on stimulus, 0) animal dead. The examiner was blinded to the animal’s treatment arm. Thereafter, the presence of hemiparesis was tested by assessment of the limb movement and forepaw stretching.

### Quantification of subarachnoid blood and histological assessment

Following neurological assessment, the animals were again anesthetized with Isoflurane. Thereafter, an intraperitoneal injection of 1 ml of sodium pentobarbital (Narcoren, Boehringer Ingelheim, Germany) was administered and the animals were transcardially perfused with 4% paraformaldehyde. The animals’ brains were removed and extent of SAH was quantified under the operation microscope using the Sugawara grading scale 20. A score ranging from 0 to 18 was given based of the amount of blood cloths in six segments of the basal cistern. Consequently SAH was classified as mild (1 – 7), moderate (8 – 12) or severe (13 – 18).

After cryo-asservation the perfused brains were cut in 16μm coronary sections using a cryomicrotome (Leica CM3050s, Leica Mikrosystems Nussloch GmbH, Heidelberg, Germany). Per animal two defined parts of the CA1 region of the hippocampus (bregma -3.72 and 4.92) were determined according to a stereotactic atlas of the brain 21. The sections were stained with a Caspase-3 antibody (Cleaved Caspase-3 (Asp175) Antibody, 1:600, Cell Signaling Technology, Inc., Danvers, Massachusetts, USA) and co-stained with DAPI (4‘,6- Diamidino-2-phenylindole, Sigma- Aldrich, St. Louis, Missouri, USA). The CA1 areas of the sections were bilaterally scanned for Caspase-3 positive cells per visual field under 40-fold magnification using a fluorescence microscope (Leica DMI 3000B, Leica Microsystems, Wetzlar, Germany). The number of damaged cells is depicted as a percentage of total visible cells in the visual field. The cell count was performed by an examiner blinded towards the animals’ treatment arms.

### Statistical analysis

Statistical analysis was performed using GraphPad Prism 4 (GraphPad Software, San Diego, California, USA). Data was tested for normal distribution using the D’Agostino and Pearson normality test. The measured end points of the experiment were compared using a one-way analysis of variance (ANOVA). When appropriate, Tukey’s test for multiple comparisons was applied. The difference in mortality between the experimental groups was analyzed by a χ^2^-test. A p-value of < 0.05 was considered significant. Results are presented as mean ± standard deviation (SD).

## Results

### Physiological parameters

Values of arterial blood gases are depicted in Table 1. There were no significant differences between the groups regarding arterial pH, pO2 and pCO _2_ throughout the experiment.

**Table 1:** Arterial blood gases before and in hourly intervals after induction of SAH. Differences were not significant throughout the monitoring time.

		MTP	MC	Control	p
Before	pH	7.37 ± 0.05	7.40 ± 0.05	7.39 ± 0.06	0.671
SAH	pO_2_	144.4 ± 24.5	132.5 ± 20.1	139.0 ± 21.0	0.734
	pCO_2_	39.7 ± 4.6	36.3 ± 8.8	44.5 ± 5.9	0.118
60 min.	pH	7.33 ± 0.05	7.36 ± 0.06	7.38 ± 0.06	0.319
	pO_2_	144.4 ± 24.5	132.5 ± 20.1	139.0 ± 21.0	0.734
	pCO_2_	42.7 ± 5.6	40.1 ± 5.3	43.1 ± 8.7	0.719
120 min.	pH	7.38 ± 0.04	7.36 ± 0.05	7.38 ± 0.05	0.543
	pO_2_	115.6 ± 24.9	108.8 ± 21.4	116.0 ± 18.2	0.904
	pCO_2_	40.7 ± 5.6	41.4 ± 3.2	43.1 ± 6.3	0.459

### Mean arterial blood pressure, intracranial pressure and cerebral perfusion pressure

**Figure 1a-c:**
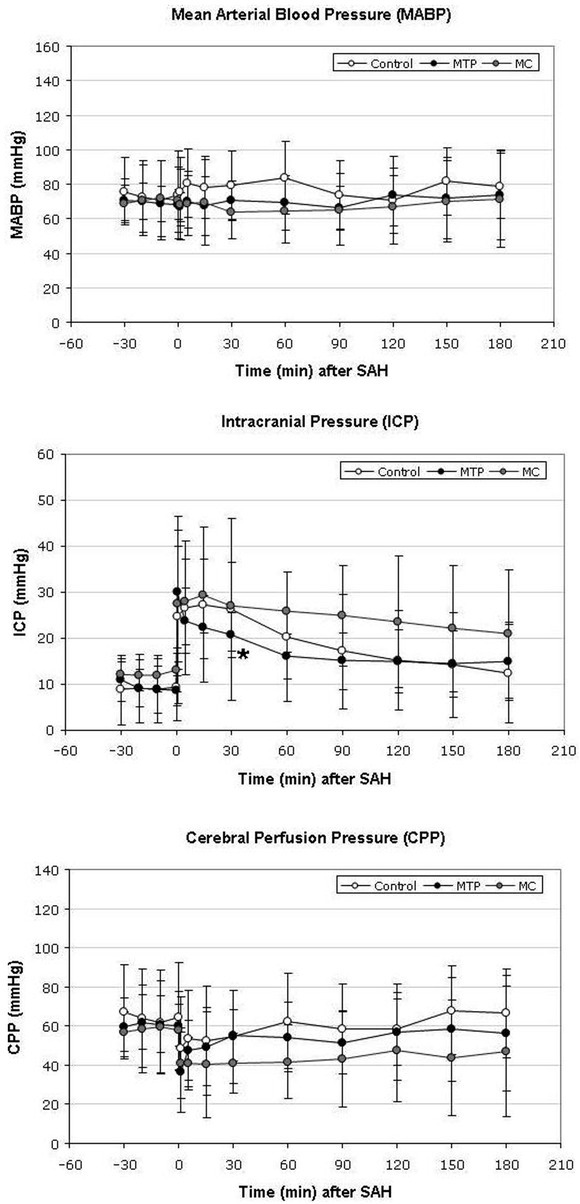
Course of mean arterial blood pressure (MABP), intracranial pressure (ICP) and cerebral perfusion pressure (CPP) continuously measured from 30 minutes before until 180 minutes after induction of subarachnoid hemorrhage (SAH). While there was no statistically significant between the groups regarding the course of MABP (1a), ICP was significantly lower in animals treated with methylprednisolone (MTP) than the other groups 30 minutes after induction of SAH. Although a strong trend is visible that animals of the minocycline (MC) group tended to higher ICP values from 60 – 180 minutes after induction of SAH, the differences were not significant (1b). Consequently, CPP tended to be lower in the MC group closely missing the level of significance (p = 0.06 at 30 and 60 minutes after SAH). The values depicted are mean ± SD. (*p < 0.05)

Immediately before the induction of SAH, MABP was 74 ± 25 mmHg, 68 ± 9 mmHg and 71 ± 17 mmHg in the control-, MTP-, and MC groups, respectively. After SAH, a slight increase of MABP was recorded to reach maximum values of 81 ± 20 mmHg and 84 ± 21 mmHg after 5 and 60 minutes, respectively. In the treatment groups, MABP remained constant slightly below 70 mmHg throughout the experiment with a minor increase at the end of the monitoring period to reach 74 ± 26 mmHg in the MTP group and 75 ± 21 mmHg in the MC group. Differences between the groups were not statistically significant (Figure 1a).

In the control group, ICP increased to a maximum of 27 ± 17 mmHg 15 minutes after induction of SAH and gradually declined to 12 ± 11 mmHg at the end of the monitoring period. In the MTP group, ICP increased to a maximum of 30 ± 17 mmHg immediately after SAH and decreased to 15 ± 8 mmHg at the end of the monitoring period.. In the MC group, ICP reached a maximum of 29 ± 8 mmHg after 15 minutes and returned to 20 ± 14 mmHg 180 minutes after the induction of SAH. Differences were not significant at any time point throughout the experiment (Figure 1b).

Baseline values of CPP were 65 ± 27, 60 ± 11 and 60 ± 20 mmHg in the control group, MTP group and MC group, respectively. CPP reached a minimum of 49 ± 26 mmHg in the control group immediately after SAH and recovered to baseline values after 60 minutes to reach a final value of 67 ± 23 mmHg after 180 minutes. In the MTP group, CPP decreased to 36 ± 26 mmHg immediately after SAH and recovered to 56 ± 29 mmHg after 3 hours of monitoring. CPP decreased to a minimum of 41 ± 18 mmHg after the induction of SAH and recovered to 47 ± 33 mmHg at the end of the monitoring period. Differences just barely missed the level of significance 30 and 60 minutes after SAH with a markedly lower CPP in the MC group compared to the control and MTP groups (Figure 1c).

### Local cerebral blood flow

**Figure 2a and b:**
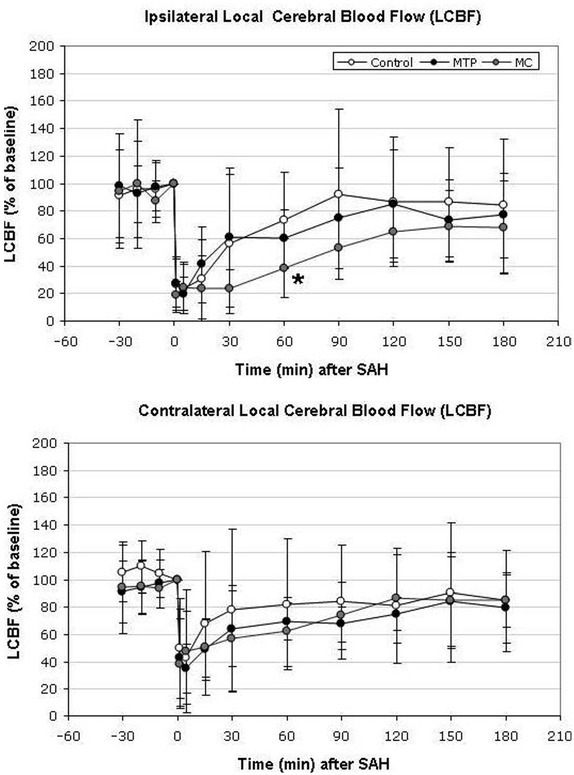
Course of local cerebral blood flow (LCBF) over the right (ipsilateral) and left (contralateral) hemisphere continuously measured by laser-Doppler flowmetry (LDF) from 30 minutes before until 180 minutes after induction of subarachnoid hemorrhage (SAH). While values in the minocycline (MC) group were lower over the ipsilateral hemisphere reaching the level of significance 60 minutes after endovascular vessel perforation (2a), there was no marked difference over the contralateral hemisphere (2b). The values are relative to baseline and depicted as mean ± SD (*p < 0.05).

In control animals, the ipsilateral LCBF declined to 27 ± 20% of baseline immediately after vessel perforation. Over the following 90 minutes, it gradually recovered to reach values between 80 and 90 % of baseline thereafter (84 ± 48 % of baseline at the end of the monitoring time). In the MTP group, LCBF dropped to a mean of 28 ± 18 % of baseline immediately after SAH and gradually recovered to 77 ± 31 % of baseline after 180 minutes. In the MC group, LCBF decreased to 18 ± 10 % of baseline after induction of SAH and slowly recovered to 68 ± 34 % of baseline after 180 minutes (Figure 2a).

In the contralateral hemisphere, LCBF dropped to 49 ± 36 % of baseline after induction of SAH and recovered to 84 ± 37 % at the end of the monitoring period. In the MTP group and MC group, LCBF declined to 43 ± 36% and 38 ± 33% of baseline, respectively and recovered to 79 ± 26% and 85 ± 19% of baseline at the end of the monitoring period, respectively (Figure 2b).

### Extent of hemorrhage, mortality, neurological performance

**Figure 3:**
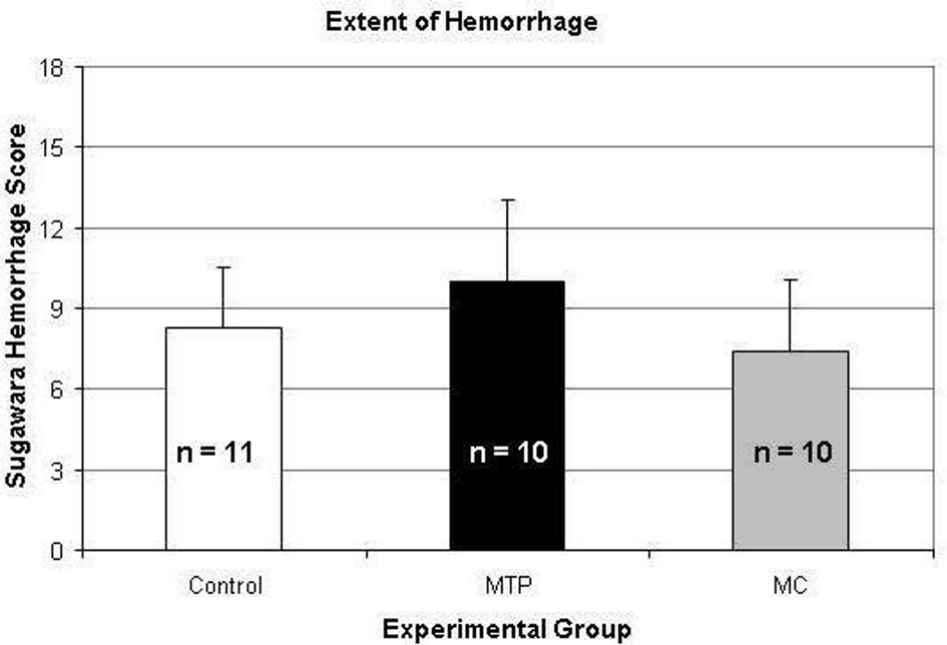
Assessment of the amount of blood in the subarachnoid space using a semiquantitative scale introduced by Sugawara et al. Differences were not statistically significant.

The average extent of SAH was 10.0 ± 3.03 in the MTP group and 8.27 ± 2.83 in the control group, classified as moderate according to the classification of Sugawara et al. The MC animals showed an average extent of hemorrhage SAH of 7.40 ± 2.69, resembling mild hemorrhage according to Sugawara’s classification (Figure 3). Differences were not significant (p = 0.16).

**Figure 4:**
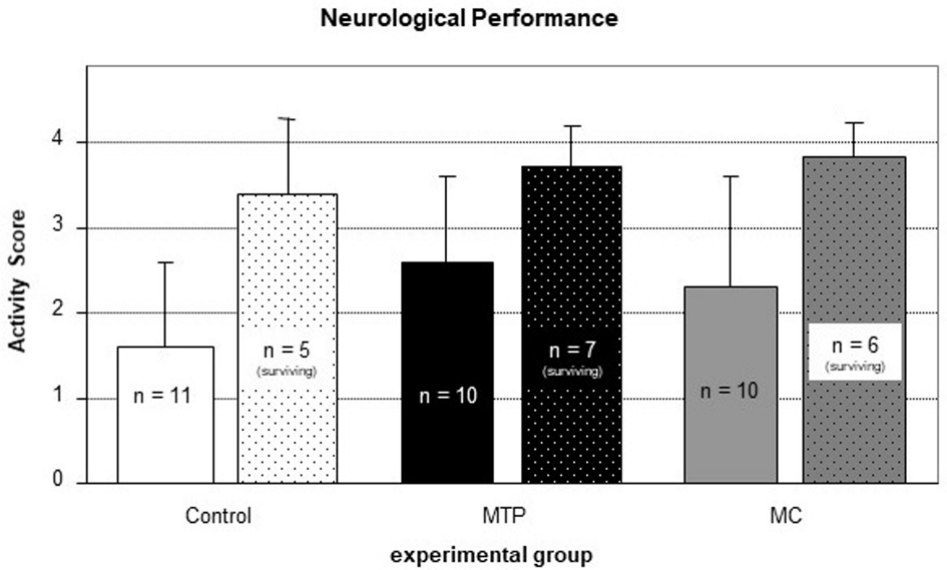
Neurological assessment 24 hours after induction of SAH by a 5-grade activity score including animals that died before the scheduled timepoint of neurological assessment (0 = dead, 4 = normal activity). These animals as zero points (Solid bars). Scattered bars indicate the neurological performance of the animals that survived the first 24 hours after SAH. Depicted are mean values ± SD.. Differences did not reach the level of significance.

Six animals of the control group, three animals of the MTP group and four animals of the MC group did not survive the first 24 hours after SAH, resulting in an overall mortality of 42%. Differences between the groups were not statistically significant. None of the surviving animals showed signs of hemiparesis as assessed by limb movement and forepaw stretching. Results of activity assessment 24 hours after SAH are depicted in Figure 4. Differences between the groups regarding the activity score did not reach the level of significance (p = 0.42).

### Histological damage

**Figure 5:**
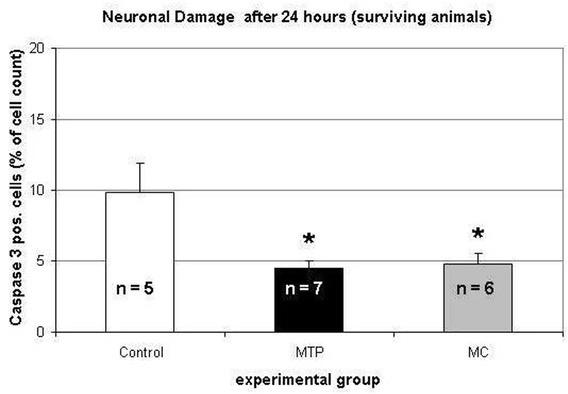
Analysis of hippocampal (CA1) damage by immunohistochemical Caspase 3 staining. Values represent the rate of caspase 3 positive cells of the total cell count per visual field under 40-fold magnification (*p < 0.05).

The percentage of Caspase 3 positive cells in the hippocampal CA1 region was analyzed in animals that survived 24 hours after SAH (n = 5 in the control group, n = 7 in the MTP group and n = 6 in the MC group). Hippocampal damage was significantly reduced in the MTP and MC group compared to the control group (Figure 5).

## Discussion

In these experiments, a well established and standardized model of experimental SAH in rats was used to assess the neuroprotective potential of MTP and MC, two drugs that have long been approved by pharmacological agencies, are readily available and have a well known safety profile. The results suggest that early treatment with anti-inflammatory drugs can reduce early brain damage and improve mortality and neurological performance after experimental SAH.

The endovascular filament model is particularly useful to assess early pathophysiological changes and to compare them with parameters of early brain damage 22. It was the particular aim of this work in the framework of a larger project to investigate whether there is a beneficial effect of treatment in the early phase of the disease. The substantial disadvantage of the endovascular perforation model is its high mortality as confirmed in the present experiments 17,23. However, compared to other studies using this

same model, pathophysiological changes were relatively mild in this series of animals 24. In particular, ICP increased only moderately, the reason for which may be a relatively low arterial blood pressure (70 – 80 mmHg) prior to the induction of SAH. Lower baseline blood pressure may result in less extravasation in the case of vessel perforation. This may explain the relatively low extent of extravasated blood in the subarachnoid space which was semiquantitatively assessed by the scoring system suggested by Sugawara et al. 20.

Although brain edema is predominantly cytotoxic in the acute stage of SAH, the early reduction of ICP in MTP-treated animals may be a short-term antiedematous effect of the corticosteroid, possibly by the inhibition of natriuresis [25, 26, 27]. Higher ICP and lower CPP and ipsilateral LCBF in the group treated with MC can hardly be explained by the pharmacological profile of the drug but rather with a limited number of 2 animals showing a particular increase of ICP after the induction of SAH. In the present sample sizes, this may result in a marked difference, however not statistically significant.

The observation that not only MTP but also MC offered a beneficial effect, although LCBF was reduced in the latter, underlines that immunological mechanisms may strongly contribute to early brain damage after SAH and their treatment in an early stage and in adequate dosage may exert a protective effect.

The pathophysiological cascade of early brain injury after SAH is complex offering a variety of possible targets for therapeutical intervention 28,29. Extravasated hemoglobin, damaged endothelial barrier and free oxygen radicals lead to the formation of brain edema and activation of an inflammatory response that ultimately enhance or result in an uncontrolled apoptosis and/or necrosis 30,31. In the last decade, the knowledge about the pathomechanisms of EBI has increased, but no early neuroprotective treatment after SAH has been introduced into clinical practice. In the present study, we investigated two anti-inflammatory drugs that have been in clinical use for many decades and are, thus ready for use.

MTP has been found to inhibit the induction of apoptosis, represses pro-inflammatory cells and mediators and the induction of suppressor cells 32. In addition, it attenuates lipid peroxidation, reduces oxidative stress and stabilizes the endothelial barrier 33.

MTP has been used previously in the management of SAH in order to prevent or treat delayed cerebral vasospasm [34, 35, 36, 37]. In a metaanalysis of 2005, though, Feigin et al. concluded that the evidence was not sufficient to support its routine use for prophylaxis of delayed cerebral ischema 38. In 2010, Gomis et al. published the results of a randomized controlled trial in which they found that early high-dose treatment with MTP resulted in a significantly improved long-term outcome after aneurysmal SAH 12. In analogy, Czorlich et al. recently reported that treatment with Dexamethasone resulted in a significant improved neurological recovery 4 months after aneurysmal SAH 39.

Similarly, MC has a wide range of anti-inflammatory and potentially neuroprotective qualities. In experimental models of various neurologic diseases, positive effects upon on apoptotic, inflammatory and oxidative signaling pathways have been observed [40, 41, 42]. These may be the result of its ability to down-regulate matrix metalloproteinase (MMP-) 9, phospholipase A2 and interleukin (IL-) 1β converting enzyme, which play a major role in inflammatory and membrane destabilizing pathways [16, 43-45]. Furthermore, the direct inhibition of cytokines like tumor necrosis factor α, interleukin 6 or interleukin 1β and the attenuation of caspase dependent and independent apoptosis are further effects of the tetracycline derivate MC [13, 14, 35, 46]. In addition, MC is a potent iron chelator forming iron complexes with the potential to attenuate the inflammatory response by the reduction of its neurotoxic stimulus 14.

Theoretically, beneficial effects of MTP could also be based on a CBF enhancing capacity via inhibition of prostaglandin synthesis 10, and leukocyte-endothelium interactions 11. Similarly, MC may stabilize CBF via inhibition of MMP-9, global microglia activation, and adhesion/diapedesis of leukocytes [45, 47, 48].

In the present study, an improvement of CBF was not observed in the treatment groups. In contrast, the treatment groups showed a tendency to lower CBF levels, suggesting that the improvement of neurological performance and attenuation of hippocampal damage are the result of intrinsic anti-inflammatory actions of those two drugs.

EBI after SAH seems to play a major role in the development of bad outcome in this patient population 49,50. In a number of studies it has been shown that inflammatory pathways, oxidative damage caused by free oxygen radicals and disruption of the blood brain barrier lead to uncontrolled apoptosis and necrosis and, therefore, may result in neuronal damage 51,52. The therapy with MTP reduced the amount of caspase 3 positive cells, a marker of apoptotic transformation, in the animals’ hippocampus. The inhibition of various mechanisms modulating inflammatory reactions like transcription factor NF kappa B 10 and lipid peroxidation are possible mechanisms responsible for this finding 33,53.

MC also reduces hippocampal damage 24 hours after SAH, as well. The neuroprotective potential of the tetracycline antibiotic has been reported for a number of different neurological disorders 54. The results of this study are in accordance with previous results obtained in experimental studies of SAH. Using a double injection model in rats, Guo and coworkers concluded that MC may also reduce EBI by inhibiting MMP-9 43. Furthermore, it may, in higher doses, reduce brain edema after SAH induced by the endovascular perforation model in rats 55. Recently, Li et al. published data that showed a reduction of apoptotic markers such as p53 and Bax as well as inflammatory target proteins like IL-1β and NLRP3 inflammasome 56.

This study has clear limitations with respect to the small sample size and the early assessment of endpoints. As stated above this series is part of a large project assessing the positive effects of a variety of drugs and mechanisms which are potentially neuroprotective considering the current knowledge about the pathophysiology of EBI. To elaborate differences on physiological parameters including CBF these sample sizes are usually adequate. These parameters were not influenced by therapy with MTP and MC, respectively. To show an improvement of neurological performance, sample sizes are too small. The main finding is the improvement of hippocampal damage after 24 hours in spite of a trend to lower CBF values. This timepoint may be relatively early but it was the distinct aim of this project to address the effects of early brain damage after SAH and not mix up with any form of delayed ischemic process. Pathophysiological changes in rodent models may proceed more rapidly than in humans. Therefore, the endpoint for neurological assessment and histological assessment was deliberately chosen.

## Conclusion

In this experimental study, an attenuation of neuronal damage has been observed by therapy with the corticosteroid MTP and the tetracycline MC. Both substances have a number of potentially neuroprotective qualities. Since physiological parameters were not improved by treatment with either substance, we conclude that the strong anti-inflammatory action is most likely to be the key factor for the beneficial effects.
